# Highly accurate machine learning prediction of crystal point groups for ternary materials from chemical formula

**DOI:** 10.1038/s41598-022-05642-9

**Published:** 2022-01-28

**Authors:** Abdulmohsen Alsaui, Saad M. Alqahtani, Faisal Mumtaz, Alsayoud G. Ibrahim, Alghadeer Mohammed, Ali H. Muqaibel, Sergey N. Rashkeev, Ahmer A. B. Baloch, Fahhad H. Alharbi

**Affiliations:** 1 Physics Department, King Fahd University of Petroleum and Minerals, Dhahran, Saudi Arabia; 2 Electrical Engineering Department, King Fahd University of Petroleum and Minerals, Dhahran, Saudi Arabia; 3 Interdisciplinary Research Center for Hydrogen and Energy Storage, King Fahd University of Petroleum and Minerals, Dhahran, Saudi Arabia; 4 Open Systems International Inc., Montreal, Quebec Canada; 5 Department of Materials Science and Engineering, University of Maryland, College Park, MD USA; 6 Research & Development Center, Dubai Electricity and Water Authority (DEWA), Dubai, United Arab Emirates

**Keywords:** Computational methods, Computational chemistry, Structure of solids and liquids

## Abstract

One of the most challenging problems in condensed matter physics is to predict crystal structure just from the chemical formula of the material. In this work, we present a robust machine learning (ML) predictor for the crystal point group of ternary materials (A$_l$B$_m$C$_n$) - as first step to predict the structure - with very small set of ionic and positional fundamental features. From ML perspective, the problem is strenuous due to multi-labelity, multi-class, and data imbalance. The resulted prediction is very reliable as high balanced accuracies are obtained by different ML methods. Many similarity-based approaches resulted in a balanced accuracy above 95% indicating that the physics is well captured by the reduced set of features; namely, stoichiometry, ionic radii, ionization energies, and oxidation states for each of the three elements in the ternary compound. The accuracy is not limited by the approach; but rather by the limited data points and we should expect higher accuracy prediction by having more reliable data.

## Introduction

In the past few years, materials science utilizing the vast accessible data through what is known as “material informatics” has witnessed a considerable growth^1–10^ when compared to other related scientific activities such as theory, experiment, and computation. This could provide novel and unusual means to breakthroughs in accelerated materials discovery and hence competitive technological developments. One of the main motivations behind such development is the fact that the existing materials data has been enriched considerably by many computational materials initiatives^11–13^ and due to the remarkable developments in supercomputers and cloud computing. This has led to the adaptation of data analytics and Machine Learning (ML) for the search of new materials either directly or combined with other computational atomic scale methods and other data analytics methods.

The data-driven transformation is not unique to materials science. Almost every sector in basic sciences and industries^14^ is going through undeniable related paradigm shift. ML provides a predictive capability that allows us to statistically “learn” from data by extracting patterns and trends^15^. Specifically, it allows correlating the inputs and responses of many complex problems without a precise knowledge of the underlying physical parameters. However, in computational sciences and applied mathematics, the majority of the community inclines toward applications while just a few focus on the foundations and fundamental developments. This raises a serious concern of “black box” implementations and stretched extrapolations^16–18^. Unfortunately, such a case is quite common.

While ML has been implemented in almost all areas of materials science, this paper focuses on crystal structure which is one of the most important characteristics of any “solid state” material. Conceptually, it should allow deriving almost all other material’s significant properties^19–21^. In principle, a crystal is uniquely characterized by its symmetry properties (determining the normalized geometry), lattice parameters, and centering. The symmetry can be effectively described by either point groups or space groups. The former has smaller configuration space in general as it keeps one point – at least – fixed. In three-dimensional (3D) space, there are 32 point groups compared to 230 space groups^22^.

Historically, non-experimental attempts to predict crystal structures started in the 1920s for simple binary ionic compounds^23–25^. Over the years, advancements in experimentation have enabled the collection of large databases. This prompted the use of structural diagrams mapping to predict the structure of crystalline material^26–33^. However, structural diagrams are heavily empirical and limited in scope. Alternatively, classification methods using ML have been adopted lately for the screening of new compounds. Many attempts have utilized experimental observations (e.g., diffraction fingerprints) besides ML^34–38^. While these efforts are important, they could be of limited practicality in situations where experimental data are absent or limited. It would be more favorable to predict geometry just from chemical composition of a material^16,37,39–41^. This is actually a long-standing problem. Here we quote what John Maddox stated in 1988^1^:

“One of the continuing scandals in the physical sciences is that it remains in general impossible to predict the structure of even the simplest crystalline solids from a knowledge of their chemical composition”

Currently, atomic-scale electronic structure calculations based on the density functional theory (DFT)^19,34,35^ are utilized to resolve this challenge as they are very accurate when it comes to geometry predictions. However, these techniques are computationally expensive which makes them practically challenging for high-throughput materials screening^19^. As an alternative, other innovative computational approaches and optimization algorithms for predicting the crystal structure were developed and utilized^21,42^.

Recently, enabled by the advances in ML and open-source data repositories, there have been attempts to provide accurate predictive models from chemical features and descriptors^43,44^. However, the achieved performance is still modest and is not entirely based on the chemical formula. To the best of our knowledge, the best reported accuracies so far are:

- Liang et. al.^44^: 69.5% - from chemical formula to Bravais lattice,
- Zhao et. al.^43^: 77.4% - from chemical formula to crystal system and space group,
- Aguiar et al.^34^: 85.2% weighted accuracy - from chemical formula to crystal system to point group using experimental crystal diffraction as input as well.

In Liang et. al.^44^ and Aguiar et al.^34^, the total accuracy is used. As discussed later, this is not the best performance measure for imbalanced data^45^. Moreover, an extensive collection of material features is used, such as Magpie^46^, which, for the most part, are redundant, offering little to no extra information with the downside of increasing the training and classification times and possibly misleading the classifier. This indicates that current ML implementations are far from optimal performance and require more to have better prediction capabilities. In many of these efforts, specific families of crystals are targeted^39,47^ , and, hence, the generality is lost.

Crystal symmetry prediction from the chemical composition and the properties of its constituent elements is a very challenging and complex ML classification problem. There are 32 point groups making the problem immensely a multi-class problem. The same chemical formula (unique input vector in ML) can crystallize in multiple phases (polymorphism); TiO$_2$, for example, can exist in the rutile, anatase, and brookite phases. This makes the problem extremely multi-label (i.e. multiple classes for the same input).

In this paper, we adopt a recently developed methodology starting from first principles calculations^7,8^. In principle, all interactions in materials are Coulombic, hence, knowing the constituent ions properties and the geometries should allow the calculation of the other properties. Herein, we have developed a general-purpose unbiased classification model using the chemical composition as an input to predict the point groups of the ternary compounds (A$_l$B$_m$C$_n$). The main objective is to develop a point group classifier based on the chemical formula only considering three complex ML challenges; namely, 1) imbalanced data for different point groups in the materials space, 2) largely multi-class nature, and 3) multi-labelity due to polymorphism of compounds. Accordingly, an efficient binary-relevance multi-class multi-label classifier model is developed. The model was trained and cross-validated using 610,759 known ternary compounds from the Novel Materials Discovery (NOMAD) repository^16,48^. The achieved robust balanced accuracy and unit normalized MCC (Matthews correlation coefficient^45^) scores are both 95%. The developed methodology was validated by using many different ML classification methods and the results show comparable high accuracies. The developed model can be used to identify the structures of unknown ternary compounds with any arbitrary stoichiometry providing that neutrality and possible oxidation states are maintained. Besides the stoichiometry, only the ionic radii, ionization energies, and oxidation states are used as input parameters.

**Figure 1:**
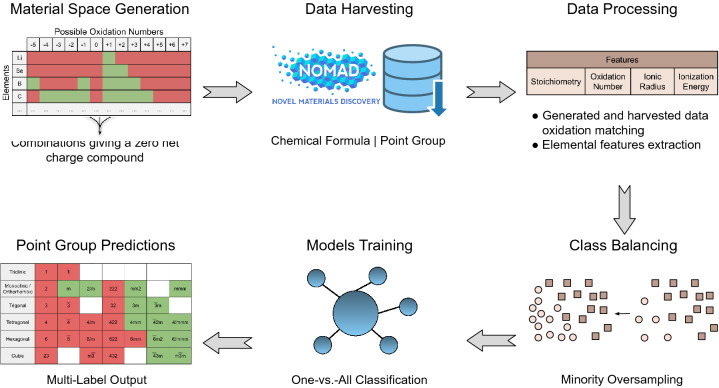
The generic workflow of point group classification from the chemical formula. It starts from the generation of the material space based on the common and uncommon oxidation states of the constituent elements. All elements of the periodic table till atomic number 85 are considered except H, Au, Pt, and noble gases. Then, the material space is matched with the harvested structural data from the open access NOMAD repository^48^. After that, the coefficient, oxidation number, ionic radius and the first ionization energy of each constituent element are processed as features for learning purposes. Next, the influence of imbalance distribution of data is mitigated using minority oversampling. Then, the model is trained using one-vs-rest classifier. Finally, the point groups of the chemical formulae are predicted which can possess more than one label.

## Methods

A multi-class, multi-label classification model is developed using binary relevance with a resampling algorithm, giving 32 binary classifiers, one for each point group. The optimized set of features includes the stoichiometry, ionic radii, first ionization potentials, and the oxidation states. The data are extracted by matching each chemical formula (for ternary compounds) in NOMAD repository with the generated material space of ternary materials, which was generated using most of the elements (up to atomic number 85) along with their possible oxidation states. The general framework is depicted in Fig. 1. The main details of the work are presented in the following subsections. The next “Material space generation” section discusses the criteria and generation of the material space. Details for the data acquisition along with the needed processing are provided in “Data harvesting, processing, and analysis” section. Then, the theoretical reasoning for the feature selection is detailed. Finally, the last subsection discusses the model development.

### Material space generation

First, a material space consisting of all the possible ternary compounds is generated using the possible oxidation states for the considered elements following the procedure proposed by Davies et al.^49^. In this work, all the elements with an atomic number of 85 and below are considered, excluding Hydrogen (H), Gold (Au), Platinum (Pt), and Group 8 elements (noble gases). Utilizing the known oxidation states for each element^50–52^, all the possible ternary compounds are generated by imposing the condition of having a final charge-neutral chemical formula. The resulting material space size is around 605 million; four times larger than what was suggested by Davies et al.^49^ as more oxidation states are considered. This huge materials space makes the task of generating and handling it a strenuous one. Furthermore, ternary compounds can exist in two possible configurations, either two of the elements are anions with the third being a cation or the other way around. For each case, the possible oxidation states were considered to obtain the charge-neutral combinations for the constituent elements.

### Data harvesting, processing, and analysis

Many materials databases were considered to collect data. However, having as large as possible consistent and reliable data is crucial for proper classification. Consequently, we found that NOMAD^48^ is the most suitable option and hence the data is harvested from it using its Application Programming Interface (API). By searching for compounds in the generated materials space, 610,759 compounds were found. In this step and for each considered chemical formula, the elements are permuted to include the 6 possibilities in case of mixed nomenclatures. In case of multiple matches, only one of the 6 permutations are kept to prevent data leakage. This is needed so that they can be matched with the acquired database compounds.

**Figure 2:**
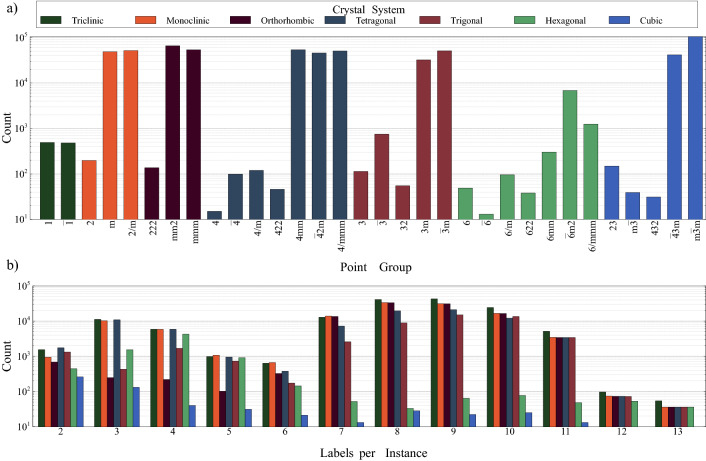
The distribution of the acquired structural data for 610,759 ternary materials. (**a**) The distribution of the structural data w.r.t the point groups where the corresponding crystal system is identified; and (**b**) The distribution of the multi-label chemical formula for every crystal system. For most of the number of instances, triclinic crystal system is the highest populated system while ternary materials with cubic crystal system are the least frequent to occur. The majority of the multi-label material space span between 7 and 11 labels per instance.

Figure 2a depicts the count of found data per point group, which shows the clear class imbalance in the data. This is expected since for a smaller number of constituent elements, the highly symmetric point groups will be more populated. So for ternary materials, point groups with higher symmetry are more populated. There are 971 ternary materials with triclinic crystal system. They are distributed approximately equally between 1 and $\overline{1}$ point groups. The population of ternary materials with monoclinic crystal system is 100,633, where *m* and 2/*m* point groups comprise the majority. For the orthorhombic crystal system, there are 119,524 ternary materials where most of them belong to the *mm*2 or *mmm* point groups. The number of ternary materials with tetragonal crystal system is 150,573 where the minority of this materials set populate the 4, $\overline{4}$, 4/*m* and 422 point groups. For trigonal crystal system, there are 83,895 ternary materials that are distributed among five point groups. Interestingly, only 8594 ternary materials exist with hexagonal crystal system where $\overline{6}m2$ point group is the most populated one. There are 146,793 ternary materials with cubic crystal system and the $m\overline{3}m$ point group is the most populated symmetry among all of the point groups of the crystal systems.

Furthermore, it is found that the obtained data has considerable multi-labelity, that is, some of the chemical compounds are polymorphic, possessing more than one possible point group symmetry. Figure 2b shows the number of times each multi-label case has occurred, where 181,054 chemical formulae are unique; i.e. compounds of the same chemical formula can crystallize differently. In extreme cases, there are 18 ternary materials found computationally to crystallize in 13 different point groups.

### Feature selection

One of the main ML step ensuring proper physical consideration and also affecting the performance of an ML model is the choice of features representing the used dataset^3^. In some cases, theory – in prior – can guide feature selection while in other cases, statistical dimensionality reduction methods or even test-and-trial approaches are used. In most cases, a combination of these methods is used. Therefore, identifying the features is the key scientific ingredient to apply ML in the physical sciences. Furthermore, it is important to pick the features in a way, which avoid underfitting, overfitting, and redundancy^3,5,6,12,37,53,54^. Interestingly, something related was proposed much before “Machine Learning” by Pauling in his seminal 1929 work^24^ on the structure of ionic crystals. He emphasized that in nature, things tend to behave in the simplest manner possible. This is known as the law of parsimony^4,24^. In crystals, the interactions are Coulombic and hence we should assume that the governing models would be essentially minimalistic^55^. Concerning redundancy, it is not uncommon to find applications of features that are proportional to each other^5,37,53–55^. For example, if one uses the atomic radius as a feature, then the atomic volume becomes redundant and using it will not help if the used regression utilizes transformations or kernels.

As the aim of this work is to predict the structure only from the knowledge of the chemical compositions, the used features must be elemental. Initially, many features were considered. The list is then reduced to eliminate apparent redundancies by considering many different techniques like the correlation factor as shown in Fig. S1 and Fig. S2 (in Supplementary Information). In these heat map figures, the absolute values of the calculated correlation coefficient are shown. Features with high correlations should not be used jointly as they would be redundant. The correlations of the final list of features are shown in Fig. S2 and it is clear that the correlations are weak between the selected features. The final reduced set of features are the ionic radius (not to be confused with the atomic radius)^56^, first ionization energy, the oxidation state of each element, and the stoichiometric coefficient. In total, the number of the used features is 12 (4 features per element).

### Development of classification model and performance indicators

**Figure 3:**
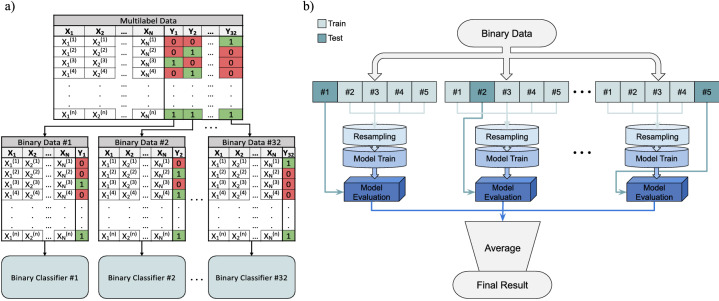
Classifier development procedure. (**a**) Multi-label data transformation to the binary relevance format. The first point group ($\mathrm {Y}_1$), is taken and fed to the binary classifier algorithm. The next binary data $\mathrm {Y}_2$ is processed using the same procedure till the last binary data $\mathrm {Y}_{32}$. If the point group cell is 0, the chemical formula at that row does not exist with this symmetry and vice versa. The configuration of each point group 0 or 1 varies among the considered material space for training; and (**b**) The implemented 5-fold cross validation approach for each binary classification problem. The testing data set varies from one fold to another. After that, the training data set is resampled and subsequently trained for each fold. Then, the training model is quantified using the unseen testing data set. Finally, the evaluation metrics of the five folds are averaged to obtain the final result.

Multi-label problems present a strenuous challenge as explained earlier; such classifiers must find a set of labels for each instance. In this work, the used approach is binary relevance, where the multi-label learning task is decomposed into multiple one-versus-all binary learning tasks which are independent, one for each point group (class label). To apply binary relevance scheme as depicted in Fig. 3a, the entries with the same chemical formula are merged to allow for independent binary classification. We end up with 181, 054 unique chemical formulae.

However, this binary relevance maneuver, from a single multi-label classification problem into 32 binary classification problems, makes the data for each binary classifier hugely skewed toward the false class. In general, the results of imbalanced data are biased toward the majority class. Therefore, the transformation from imbalanced to balanced data is essential, especially for the extremely low count classes. In this work, the Synthetic Minority Over-sampling TEchnique (SMOTE), which was proposed by Chawla et al.^57^, is used to equalize the counts of the two binary classes of the training data of each classifier. Figure 3b illustrates the developed algorithm, where a 5-fold cross-validation is used to better assess the validity of each of the 32 binary classifiers. The implemented folding strategy is stratified, that is, the true classes are equally distributed to each fold. Many classification methods are used and the majority of them result in excellent and comparable accuracies. In all cases, the default hyperparameters were kept as is without tuning to avoid undermining the fidelity and validity of the obtained scores. To evaluate the accuracy of the developed point group classifier, the Balanced Accuracy (BA) and the unit normalized MCC (UM)^45^ are utilized based on the genuine test sets (no synthetic data are used for testing). For imbalanced data as in our case, the standard accuracy and F-measure (F$_1$) profoundly exaggerate the optimistic statistical measures and produce inflated results. More details and comparisons of classification metrics are provided in the Supplementary Materials. BA and UM are defined as follows:

1 $$\begin{aligned} \text {BA}=\frac{1}{2} \left( \text {Sensitivity} + \text {Specificity} \right) = \frac{1}{2} \left( \frac{\text {TP}}{\text {TP+FN}} + \frac{\text {TN}}{\text {TN+FP}} \right) \end{aligned}$$

and

2 $$\begin{aligned} \text {UM}=\frac{1}{2} \left( 1 + \text {MCC} \right) = \frac{1}{2} \left( 1 + \frac{\text {TP}\cdot \text {TN}-\text {FP}\cdot \text {FN}}{\sqrt{(\text {TP}+\text {FP})\cdot (\text {TP}+\text {FN})\cdot (\text {TN}+\text {FP})\cdot (\text {TN}+\text {FN})}} \right) \end{aligned}$$

where TP is true positive, TN is true negative, FP is false positive, and FN is false negative for binary classifiers. Both BA and UM range between 0 and 1 where 1 corresponds to perfect classification.

BA and UM are single binary classifier measures. For multi-label multi-class problems, the resulting measures are a set of vectors. Thus, other global means are used to assess the classifications of such multi-label multi-class problems. Weighted averages are adopted in this work to account for the fact that particular point groups are more probable than others. The used measures are the Weighted Balance Accuracy (WBA)

3 $$\begin{aligned} \text {WBA}=\frac{1}{N_p} \sum _{g} N_g \, \text {BA}_g \end{aligned}$$

and the Weighted Unit normalized MCC (WUM)

4 $$\begin{aligned} \text {WUM}=\frac{1}{N_p} \sum _{g} N_g \, \text {UM}_g \end{aligned}$$

where $N_g$ is the number of positive count in the $g^\text {th}$ point group and $N_p$ is the sum of all $N_g$’s. Also, to ensure that the classification is not biased toward neither the majority nor the minority classes, another measure, the Balance (B) is used^58^, which is the ratio between the specificity and sensitivity. The value of B should be around 1; otherwise, the classification is not balanced and it inclines toward one of the classes. For each point group classifier, B is

5 $$\begin{aligned} {\text {B}}_g=\frac{{\text{Sensitivity }}}{{\text{Specificity}}}= \frac{{\text{TP}}}{{\text{TP}}+{\text{FN}}} \, \frac{{\text{TN}}+{\text{FP}}}{{\text{TN}}} \end{aligned}$$

and the Weighted Balance (WB) is

6 $$\begin{aligned} \text {WB}=\frac{1}{N_p} \sum _{g} N_g \, \text {B}_g \, . \end{aligned}$$

## Results and discussion

### Classification reliability

In ML, it does not suffice to develop an accurate predictive model^18^. The model must be reliable, reproducible, and testable. The main challenge is how to assess such aspects^9,15,17,18,59^. One of the common practices to assess reliability is to use multiple independent ML techniques. The models can be assumed reliable if many of them produce comparable results. Thus, different classification methods are used either solely or in a hybrid manner. The used methods^60^ are:

1. *k*-nearest neighbors (KNN),
2. Decision tree (DT).
3. Random forest (RF),
4. Bootstrap aggregating (BG),
5. Gaussian naive Bayes (GNB),
6. Linear models,
7. Linear discriminant analysis (LDA).

**Figure 4:**
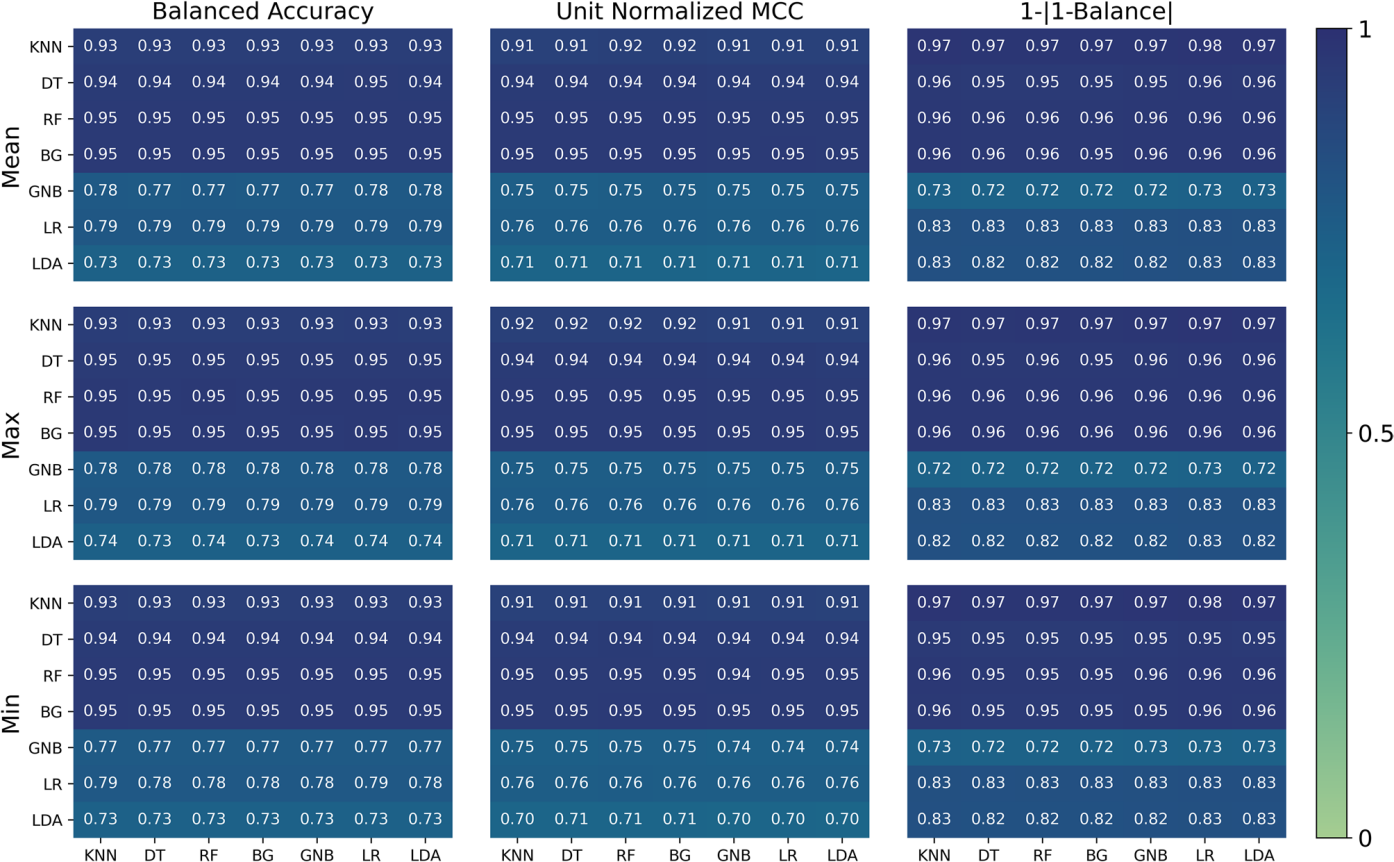
The obtained classification measures for the considered 49 sole and hybrid classification schemes. The main columns from left to right correspond to BA, UM, and $1-\left| \text {B}-1 \right|$. On the other hand, the rows correspond to the weighted cross-validated mean, best, and worst models. The point groups are divided into two sets depending on whether they have a high positive count or low positive count. The threshold is 19K positive counts, which represents 1% of the total number data. The vertical (horizontal) axis refers to the classifier applied on the high (low) positive count set. For each panel, the diagonal cells illustrate the scores using a single type of classifier for both sets while the off-diagonal cells consist of the scores from a distinct pair of classifiers.

In these implementations, the binary classification problems are grouped into two sets; one for the sets have very low positive counts (LPC) ($< \frac{N_{{\rm Total}}}{32} \approx 19{,}086$ element per classifier) where $N_{\text {Total}}$ is the total number of data points (610,759) while the second for the high positive counts (HPC) contains all the others. Then, independent classification approaches are used for each set. This results in 49 sole and hybrid possible classification schemes. The results are summarized in Fig. 4. The columns from left to right correspond to BA, UM, and alternative transformation of the balance as $1-\left| \text {B}-1 \right|$. The balance is represented in this form to be between 0 and 1 (for most cases) where 1 corresponds to a complete balance. The rows correspond to the weighted averages of the cross-validation models mean, best, and worst performances, respectively. In all of these panels, the vertical axis corresponds to the high count classification approach while the horizontal axis corresponds to the low count one. To simplify the notation hereafter, the panel in the $i^{\text {th}}$ row from the left and $j^{\text {th}}$ column from the top is denoted as P$_{ij}$.

Clearly, most of the obtained accuracies are above 90% with maximum a WBA of 95% and WUM of 95% as shown in P$_{11}$ and P$_{12}$ obtained using Random Forest and Bagging for the high count point groups. The method used for low counts does not noticeably affect the performance. This is due to the weighting average, which gives more weight to high count point groups (the details for each group is presented and discussed shortly). Furthermore, it is clear that “similarity”-based methods like KNN, DT, RF, and BG result in excellent and comparable results unlike model-based method like GNB, LR, and LDA. However, in this work, we avoid optimizing the standard parameters of these latter methods although using proper kernels shall improve their performances. Concerning the best individual group performance, it is 99% for both BA and UM using BG, RF, and DT; while KNN is slightly lower with 96% accuracy. However, BA and UM of low count groups are much lower; this is mainly due to variance learning error (prediction fluctuation due to the training dataset) as will be discussed shortly. Their performances shall be considerably improved by having more reliable data points.

**Figure 5:**
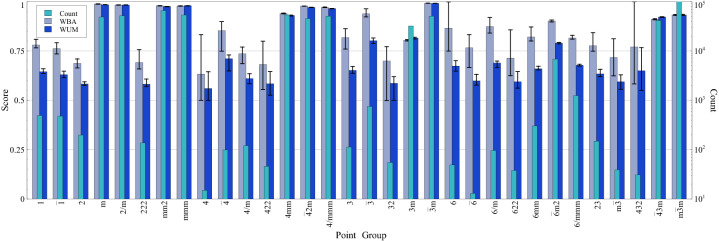
The scores of the best classification scheme and the count of each point group. The weighted balanced accuracy and the unit normalized MCC of the best performance are observed with using a pair of classifiers. RF is used for the high positive count while KNN is used for the low positive count. In the case of high positive count, the scores are very close to each other and above 90% except for point group 3*m*. On the other hand, scores span between 50% and slightly above 85% for the low positive count. Moreover, the weighted balanced accuracies surpass the weighted unit normalized MCC by approximately 10%.

However, with such big variation in the populations of the classes, the balance becomes important. The third column in Fig. 4 shows that the balance is slightly dictated by the classifier used for the low count groups. It turns out that KNN and linear classifications perform better than others for low count groups. Bagging is badly performing for the low count groups. Overall, the best performance is achieved when using Bagging for the high count groups and KNN for the low count ones, while BG performs badly. In this case, WBA=95%, WUM=95%, WB=0.94 with maximum and minimum BA for individual groups of 99% and 62%, respectively, as shown in Fig. 5. Nonetheless, many other sole and hybrid combination methods scores are almost the same which suggests the robustness of the presented approach as many classification methods result in comparable high accuracies.

### The best model

**Figure 6:**
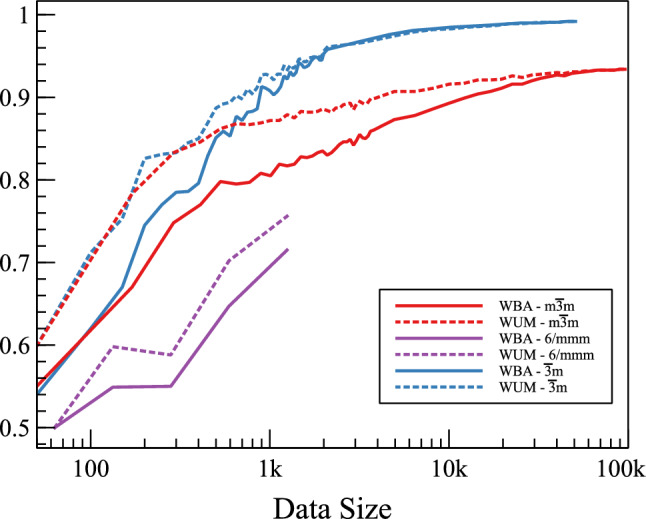
The data size dependency of the classification model. The effect of the data size is evaluated using the weighted balanced accuracy and the unit normalized MCC for the cubic ($m\overline{3}m$ point group), hexagonal (6/*mmm* point group) and trigonal ($\overline{3}m$ point group) crystal systems. The scores of the weighted unit normalized MCC are better than the weighted balance accuracy and saturate faster for all point group symmetries. The metrics become closer at saturation region and vary from one symmetry to another. Moreover, the $\overline{3}m$ and $m\overline{3}m$ point groups maintain approximately equal scores in the region of very small data size to a certain point then they split. The curves of the $\overline{3}m$ point group are the steepest and highest ones as they attain saturation (close to 1) after approximately 2 thousand data points. For the $m\overline{3}m$ point group, data points beyond 20 thousand result in both metrics exceeding 0.9. Finally, the data of 6/*mmm* point group are not sufficient to elucidate the full picture, however, 6/*mmm* point group is the lowest scoring symmetry and demands more data to reach at least beyond 0.8 values.

Figure 5 shows the obtained BAs and UMs of the best model (RF for large count and KNN for low count) along with $N_g$ for each point group. As expected, it is found that the performance of each point group classifier increases with increasing $N_g$ (see Fig. 6). This is a known fact in ML that the variance error decreases with increasing number of data points. Figure 6 presents the obtained classification BA and UM for $m\bar{3}m$, 6/*mmm* and $\bar{3}m$ point groups vs. the used positive data points. Clearly, both BA and UM increase with $N_g$. In these calculations, for every point, a completely new set is picked randomly from the main data. This resulted in the small fluctuations. Yet, the general trends are as expected. They saturate at some minimum critical number of positive data points. $\bar{3}m$ saturates at around 99% after $\sim 10,000$ positive data points while $m\bar{3}m$ needed almost 20,000 points to start saturating around 93% accuracy. In both cases, the saturation accuracy is then limited by the bias; i.e. the error due to projecting the actual physical vector space in the used ML “approximating” vector space. This is physically governed by the used features (and the used classification method). With such high accuracies, it is clear that the small set of used features (4 per elements; namely oxidation state, stoichiometry, ionic radius, and ionization energy) captures the governing physics. For the 6/*mmm* point group, the number of available positive data points is much lower. However, it is clear that the accuracy is increasing with $N_g$. It is expected that by having more reliable data, the performance shall improve. In summary, the physics is well captured by the used set of features; to improve the accuracy, we need just to reliably increase the size of the dataset. However, it should be expected that the imbalance remains almost the same as point groups with higher symmetry are naturally more populated.

## Conclusion

Inspired by the noticeable current progress of the data-driven sciences, a surrogate model is developed to predict crystal point group of ternary compounds using machine learning. In this work, we present a robust machine learning method to predict the crystal point group of ternary materials (A$_l$B$_m$C$_n$) and with very small set of needed ionic and positional fundamental features. This is first step to predict the structure just from the chemical formula, which is one of the long-lasting problems in condensed matter. From ML perspective, the problem is very challenging due to multi-labelity (polymorphism), multi-class (32 classes), and data imbalance.

The presented multi-class multi-label classification model is developed using binary relevance with resampling algorithms, giving 32 binary classifiers, one for each point group. The data are extracted by matching each chemical formula (for ternary compounds) in NOMAD repository with the generated material space of ternary materials, which was generated using most of the elements (up to atomic number 85) along with their possible oxidation states. The total number of the possible elemental combinations has surpassed 600 million materials. After that, the point group of 610,759 ternary compounds is acquired from the NOMAD open-source data repository using the chemical formula of the ternary compounds as an input criterion. The resulted prediction is very reliable and robust as high balanced accuracies are obtained by different ML classification methods. Many similarity-based methods (Bagging, Random Forest, Decision Tree) resulted in excellent performances with a balanced accuracy above 95%. Other methods as well resulted in comparable results indicating that the physics is well captured by the reduced set of features; namely, stoichiometry, ionic radii, ionization energies, and oxidation states for each of the three elements in the ternary compound. The accuracy is not limited by the approach; but rather by the limited data points for some low symmetry point groups, i.e. by having more reliable data, we should expect higher accuracy prediction.

More details concerning features selection are presented as Supporting Information.

## Supplementary Information

Supplementary Information.
