# Supplementary material for: Highly accurate machine learning prediction of crystal point groups for ternary materials from chemical formula
Source: Sci Rep. 2022 Jan 28;12:1577. doi: 10.1038/s41598-022-05642-9 (PMC8799685; doi:10.1038/s41598-022-05642-9)
Supplement: Supplementary file 1 — Supplementary Information. [file 41598_2022_5642_MOESM1_ESM.pdf]

Supplementary Material: Highly Accurate Machine Learning Prediction of Crystal Point Groups for Ternary Materials from Chemical Formula

Abdulmohsen Alsaui[1,2], Saad M. Alqahtani[3], Faisal Mumtaz[4], Ibrahim G. Alsayoud[2], Mohammed Alghadeer[1,2], Ali H. Muqaibel[2], Sergey N. Rashkeev[5], Ahmer A.B. Baloch[6], and Fahhad H. Alharbi[2,*]

[1]Physics Department, King Fahd University of Petroleum and Minerals, Dhahran, Saudi Arabia
[2]Electrical Engineering Department, King Fahd University of Petroleum and Minerals, Dhahran, Saudi Arabia
[3]Interdisciplinary Research Center for Hydrogen and Energy Storage, King Fahd University of Petroleum and Minerals, Dhahran, Saudi Arabia
[4]Open Systems International Inc., Montreal, Quebec, Canada
[5]Department of Materials Science and Engineering, University of Maryland, College Park, MD USA
[6]Research & Development Center, Dubai Electricity and Water Authority (DEWA), Dubai, United Arab Emirates
[*]e-mail: fahhad.alharbi@kfupm.edu.sa

ABSTRACT

One of the challenging problems in condensed matter is to predict crystal structure just from the chemical formula. In this work, we present a robust machine learning (ML) predictor for the crystal point group of ternary materials ($A_lB_mC_n$) – as first step to predict the structure – with very small set of ionic and positional fundamental features. From ML perspective, the problem is strenuous due to multi-labelity, multi-class, and data imbalance. The resulted prediction is very reliable as high balanced accuracies are obtained by different ML methods. Many similarity-based approaches resulted in a balanced accuracy above 95% indicating that the physics is well captured by the reduced set of features; namely, stoichiometry, ionic radii, ionization energies, and oxidation states for each of the three elements in the ternary compound. The accuracy is not limited by the approach; but rather by the limited data points and we should expect higher accuracy prediction by having more reliable data.

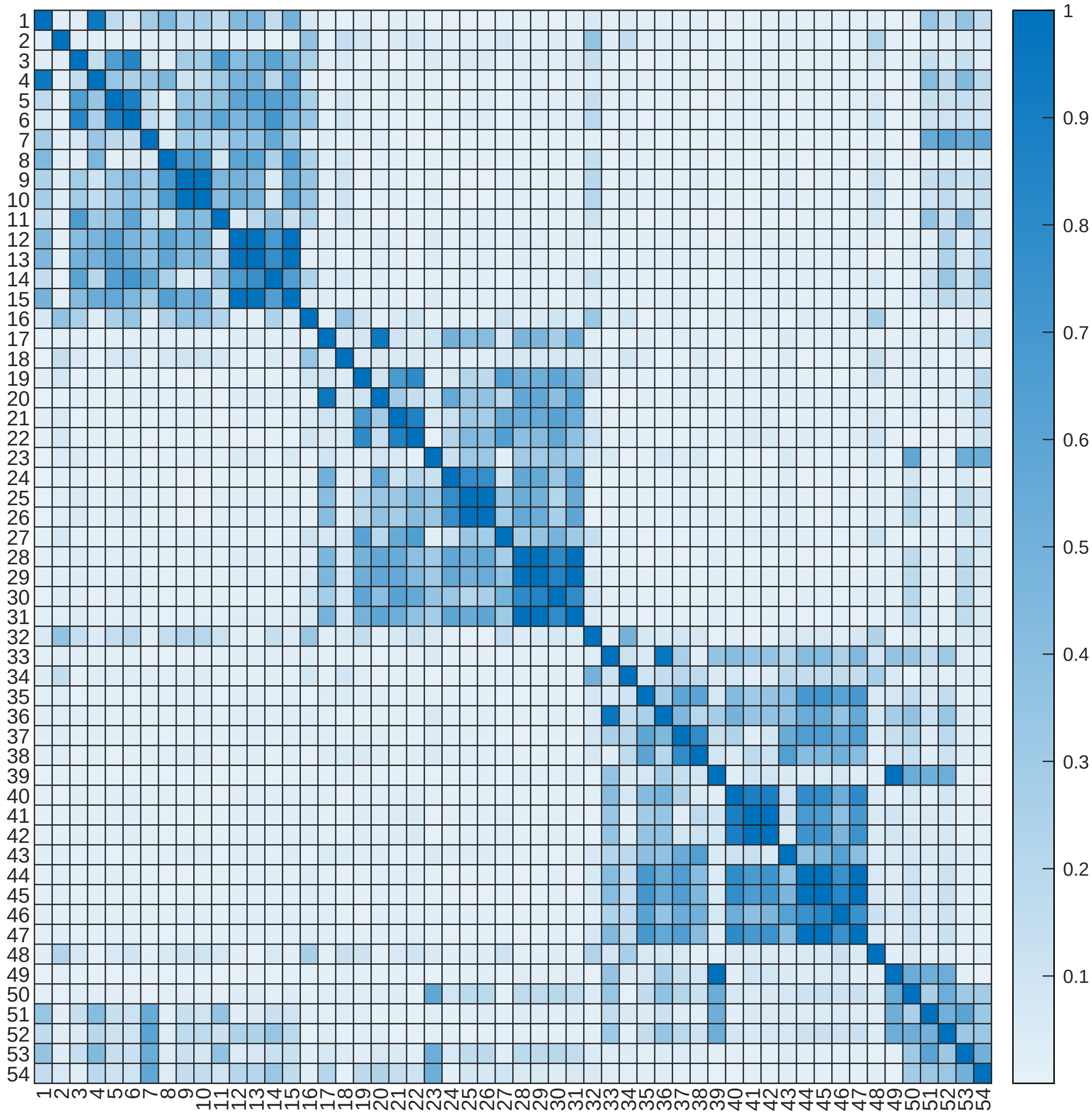

Figure S1. The calculated absolute values of the correlation coefficients of all features of all the used data. For each element in a ternary compound, 16 features (listed in the same order in the correlation matrix) are used; namely atomic number, stoichiometry, periodic table group and period, first ionization energy (first estimations), electronegativity (first estimations), ionic radius, orbitals radii (d, p, and s), energies of the lowest-unoccupied and highest-occupied Kohn-Sham (KS) levels, electronegativity (second estimations), electron affinity, first ionization energy (second estimations), and oxidation state. The last 6 features are ratios between the ionic radii of the three ions in the ternary.

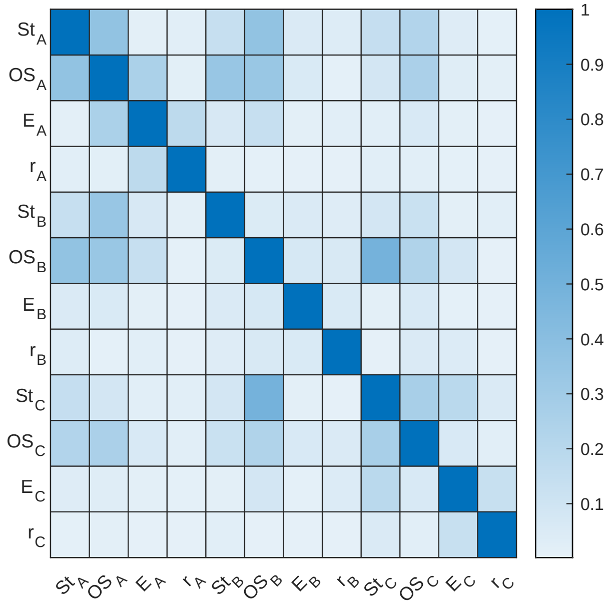

Figure S2. The calculated absolute values of the correlation coefficients of the reduced set of features of all the used data. The considered features for each of the three elements of the ternary are stoichiometry (St_i), oxidation state (OS_i), first ionization energy (E_i), and ionic radius r_i.

1 Evaluation Metrics

The confusion matrix elements incorporate all the scenarios that arise during a classification problem. Four possible elements (True positive TP, True Negative TN, False Positive FP, and False Negative FN) define the standard binary classification metrics in binary classification problems, as shown in Fig. S3.

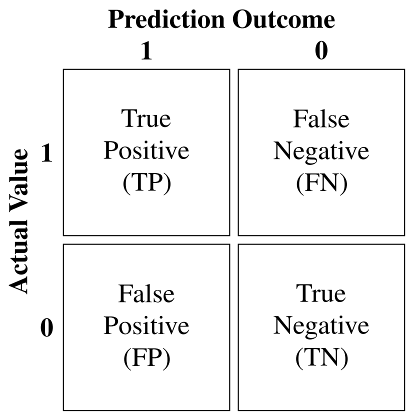

Figure S3. Binary Classification Confusion Matrix.

The most known evaluation metric is accuracy, which gives the fraction of the instances correctly classified from the total predictions. In terms of the confusion matrix elements, the accuracy is expressed as follows:

$$\text{Accuracy} = \frac{\text{TP}+\text{TN}}{\text{TP}+\text{TN}+\text{FP}+\text{FN}}, \tag{1}$$

which is biased towards the majority class in imbalanced learning[1]. This should be acceptable in data-balanced 2-class problems. Another measure is the Specificity, or true negative rate, which gives the fraction of the instances correctly classified as negative

from the total actual negatives. On the other hand, Sensitivity, or true positive rate, represents the fraction of the cases correctly classified as positive from the actual positives. The Specificity and Sensitivity can be expressed as:

$$\text{Specificity} = \frac{\text{TN}}{\text{TN} + \text{FP}}, \tag{2}$$

$$\text{Sensitivity} = \frac{\text{TP}}{\text{TP} + \text{FN}}. \tag{3}$$

Clearly, the Specificity ignores FN while the Sensitivity ignores FP. To have a measure considering the negative and positive scores equally, the Specificity and the Sensitivity can be jointly expressed using their average, defined as the balanced accuracy (BA):

$$\text{BA} = \frac{\text{Specificity} + \text{Sensitivity}}{2}, \tag{4}$$

which is a suitable metric for imbalanced learning since it considers both the positive and negative classification outcomes without being misled by the imbalance in the dataset.

Another standard metric is the precision, also known as the positive predictive value, which represents the fraction of the instances correctly classified as positive from the total predicted positives:

$$\text{Precision} = \frac{\text{TP}}{\text{TP} + \text{FP}}, \tag{5}$$

which along with the Sensitivity, define the F_1 metric as their harmonic mean:

$$F_1 = 2 \cdot \frac{\text{Precision} \cdot \text{Sensitivity}}{\text{Precision} + \text{Sensitivity}}. \tag{6}$$

Both F_1 and BA can be used for imbalanced data classification problems. However, when the negative class is the majority in the dataset and minimizing the FP is desired, BA is preferred over the F_1.

Recently, another metric that is found to be useful for imbalanced classification problems is the Matthews correlation coefficient (MCC)[1,2], which is defined as follows:

$$\text{MCC} = \frac{\text{TP} \cdot \text{TN} - \text{FP} \cdot \text{FN}}{\sqrt{(\text{TP} + \text{FP}) \cdot (\text{TP} + \text{FN}) \cdot (\text{TN} + \text{FP}) \cdot (\text{TN} + \text{FN})}}. \tag{7}$$

The MCC metric is not new but has started becoming one of the primary measures in classification problems. MCC is ranged between -1 and 1, to have a comparative range to accuracy, it is normalized to what is known as unit-normalized MCC (UM) such that:

$$\text{UM} = \frac{1}{2}(1 + \text{MCC}). \tag{8}$$

Finally and to give equal importance to the positive and negative classes prediction, the Balance (B) is introduced, which is defined by the ratios of Sensitivity and Specificity:

$$\text{B} = \frac{\text{Specificity}}{\text{Sensitivity}}. \tag{9}$$

It should be around one. If $B \gg 1$, the positive class prediction is much worse that the prediction of the negative ones and vice versa if $B \ll 1$.

References

1. Chicco, D. & Jurman, G. The advantages of the matthews correlation coefficient (mcc) over f1 score and accuracy in binary classification evaluation. *BMC genomics* **21**, 1–13 (2020).
2. Matthews, B. W. Comparison of the predicted and observed secondary structure of t4 phage lysozyme. *Biochimica et Biophysica Acta (BBA)-Protein Struct.* **405**, 442–451 (1975).
